# Editorial: Adipose tissue dysfunction

**DOI:** 10.3389/fendo.2022.999188

**Published:** 2022-08-17

**Authors:** Matthias Blüher, Dirk Müller-Wieland

**Affiliations:** ^1^ Helmholtz Institute for Metabolic, Obesity and Vascular Research (HI-MAG) of the Helmholtz Zentrum München at the University of Leipzig and University Hospital, Leipzig, Germany; ^2^ Medical Department III – Endocrinology, Nephrology, Rheumatology, University of Leipzig Medical Center, Leipzig, Germany; ^3^ Department of Cardiology, Angiology and Intensive Care Medicine, University Hospital Aachen, Aachen, Germany

**Keywords:** adipose tissue, obesity, adipokine, inflammation, lipodystrophy, visceral fat

For a long time, adipose tissue (AT) has been regarded as a functionally and anatomically simple to understand organ that only contributes to the insulation of the body, thermoregulation, and mechanical organ protection (1–3). Only in the past few decades, it has been recognized that AT is characterized by dynamic adaptations, heterogeneous cell populations, and astonishing plasticity (2–5). With the discovery that adipocytes secrete leptin (6) it became clear that AT is involved in many biological processes including regulation of energy homeostasis, glucose and lipid metabolism, blood pressure, and immune response (1–4).

With the growing interest in better understanding mechanisms causing obesity and obesity-related cardio-metabolic diseases and mortality (7, 8), AT research developed rapidly. Indeed, AT represents the main organ for energy storage under conditions of caloric surplus and releases lipids to protect our bodies against weight loss in prolonged periods of fasting or starvation. Both access to AT in people living with obesity (3, 4, 9), but also deficiency or a complete loss of AT in patients with lipodystrophy or lipoatrophy are associated with conditions that predispose to insulin resistance, type 2 diabetes, fatty liver disease, and other metabolic abnormalities (10, 11). Based on these observations, it has been proposed that impaired expandability of “healthy” subcutaneous AT may be a relevant mechanism linking obesity to its comorbid conditions (12). Supporting this hypothesis, it has been shown that transplantation of AT with a normal function led to beneficial effects on glucose and lipid metabolism in animal models (13).

The risk for many obesity-associated diseases increases linearly with an increase in fat mass (9). However, there are people with obesity who do not develop metabolic or cardiovascular diseases at the expected frequency or age of manifestation. This subgroup of people with metabolically healthy obesity has taught us about potential mechanisms for the link between fat accumulation and cardio-metabolic risk (14, 15). Among the biological mechanisms that link increased body fat mass to cardio-metabolic diseases, AT dysfunction is a major determinant of the individual obesity-associated risk (14, 15).

The concept of AT dysfunction (16) is not novel and has been described as adiposopathy (17), obesity-related adipose tissue disease (OrAD, 18), or sick fat (17). These terms summarize specific symptoms that are associated with abnormal AT function including ectopic fat deposition in visceral depots, the liver, pancreas, and other organs, adipocyte hypertrophy, a higher number of immune cells in AT, AT fibrosis, and secretion of pro-inflammatory, diabetogenic, and atherogenic signals from AT (4, 16, 17).

In principle, AT dysfunction can develop if AT in healthy subcutaneous (e.g. leg and hip fat) depots exceed their storage capacity or have (e.g. genetically determined) limited expandability. The inability of AT to respond to excess energy storage demands with adequate hyperplasia leads to hypertrophy of adipocytes that contributes to hypoxia, inadequate vascularization, AT stress and immune cell infiltration, autophagy, apoptosis, increased production of pro-fibrotic extracellular matrix proteins subsequently causing AT fibrosis.

Current AT research focuses on a better understanding of these mechanisms and specific factors with the ultimate vision to identify targets for the future treatment of obesity and AT dysfunction-related diseases. Within this Research Topic on “*Adipose tissue dysfunction*”, the complexity and fascination of AT research are reflected by eleven articles:

Pincu et al. summarize the current knowledge on histopathological characteristics of AT dysfunction and propose the term obesity-related adipose tissue disease (OrAD) to reflect the notion that abnormalities in AT of people with obesity predict future obesity-related endpoints and the response to specific anti-obesity interventions. The authors focus on adipocyte hypertrophy, AT inflammation, and fibrosis as major features of OrAD.

Our readers can find a complimentary review on immune cell regulation of white adipose progenitor cells by Altun et al. The mini-review discusses the heterogeneity in composition and fate of adipose progenitor subtypes and their interactions with different immune cell populations. More specifically, the review article by Zhu and Liu focuses on the role of activated NOD-like receptor family 3 (NLRP3) inflammasome and its interaction with autophagy mechanisms in the development of AT dysfunction.

The importance of AT immune cell infiltration in the context of AT dysfunction has been further elucidated in an original article from Strand et al. The group used a proteomics screen to identify novel surface proteins specific to M1-like- and M2-like macrophages that are fat-depot specifically associated with AT immunophenotypes.

In a sophisticated magnetic resonance imaging study, Zhang et al.support the concept that visceral AT, hepatic fat, pancreatic fat, and preperitoneal AT are associated with cardio-metabolic risk factors independently of BMI.

As lipodystrophy is considered a disease caused by AT dysfunction, our special issue includes an original article by Dattilo et al. in which the authors discovered a predictive role of circulation miRNA-320 for AT function in patients with lipodystrophy.

Ronningen et al. provide novel data on the role of N^6^-methyladenosine (m6A) - as one of the most abundant post-transcriptional modifications on mRNA – on fat depot determination.

In a large study of 5,821 adults, Sun et al. (reported that insulin resistance of AT is associated with hyperuricemia, pointing toward a previously unrecognized role AT in uric acid metabolism. The article collection is completed by novel data on associations between obesity subphenotypes and a higher risk of ischemic stroke (Zou et al.) as well as on subcutaneous fat accumulation and increased urinary stone risk in young people (Ye et al.).

We hope that you as our readers enjoy reading our Research Topic on adipose tissue dysfunction and agree with us that AT is a treasure box to identify mechanisms and targets for a better understanding of diseases that involve AT dysfunction.

## Author contributions

All authors listed have made a substantial, direct, and intellectual contribution to the work and approved it for publication.

## Conflict of interest

MB received honoraria as a consultant and speaker from Amgen, AstraZeneca, Bayer, Boehringer-Ingelheim, Lilly, Novo Nordisk, Novartis and Sanofi. DM-W received honoraria as a consultant and speaker from Amarin, Amgen, AstraZeneca, Bayer, Boehringer-Ingelheim, Daichii-Sankyo, Lilly, Novo Nordisk, Novartis and Sanofi.

## Publisher’s note

All claims expressed in this article are solely those of the authors and do not necessarily represent those of their affiliated organizations, or those of the publisher, the editors and the reviewers. Any product that may be evaluated in this article, or claim that may be made by its manufacturer, is not guaranteed or endorsed by the publisher.
